# Remote Assessment of Lung Disease and Impact on Physical and Mental Health (RALPMH): Protocol for a Prospective Observational Study

**DOI:** 10.2196/28873

**Published:** 2021-10-07

**Authors:** Yatharth Ranjan, Malik Althobiani, Joseph Jacob, Michele Orini, Richard JB Dobson, Joanna Porter, John Hurst, Amos A Folarin

**Affiliations:** 1 Department of Biostatistics and Health Informatics Institute of Psychiatry, Psychology and Neuroscience King’s College London London United Kingdom; 2 Royal Free Campus, University College London Respiratory University College London London United Kingdom; 3 Department of Radiology University College London Hospital London United Kingdom; 4 Centre for Medical Image Computing, University College London Respiratory University College London London United Kingdom; 5 Barts Health NHS Trust London United Kingdom; 6 Barts Heart Centre University College London Hospitals London United Kingdom; 7 NIHR Biomedical Research Centre at South London and Maudsley NHS Foundation Trust and King’s College London London United Kingdom; 8 Institute of Health Informatics University College London London United Kingdom; 9 NIHR Biomedical Research Centre at University College London Hospitals, NHS Foundation Trust London United Kingdom; 10 Respiratory Medicine, Division of Medicine Faculty of Medical Sciences University College London London United Kingdom

**Keywords:** mHealth, COVID-19, mobile health, remote monitoring, wearables, internet of things, lung diseases, respiratory health, mental health, cardiopulmonary diseases

## Abstract

**Background:**

Chronic lung disorders like chronic obstructive pulmonary disease (COPD) and idiopathic pulmonary fibrosis (IPF) are characterized by exacerbations. They are unpleasant for patients and sometimes severe enough to cause hospital admission and death. Moreover, due to the COVID-19 pandemic, vulnerable populations with these disorders are at high risk, and their routine care cannot be done properly. Remote monitoring offers a low cost and safe solution for gaining visibility into the health of people in their daily lives, making it useful for vulnerable populations.

**Objective:**

The primary objective is to assess the feasibility and acceptability of remote monitoring using wearables and mobile phones in patients with pulmonary diseases. The secondary objective is to provide power calculations for future studies centered around understanding the number of exacerbations according to sample size and duration.

**Methods:**

Twenty participants will be recruited in each of three cohorts (COPD, IPF, and posthospitalization COVID). Data collection will be done remotely using the RADAR-Base (Remote Assessment of Disease And Relapse) mobile health (mHealth) platform for different devices, including Garmin wearable devices and smart spirometers, mobile app questionnaires, surveys, and finger pulse oximeters. Passive data include wearable-derived continuous heart rate, oxygen saturation, respiration rate, activity, and sleep. Active data include disease-specific patient-reported outcome measures, mental health questionnaires, and symptom tracking to track disease trajectory. Analyses will assess the feasibility of lung disorder remote monitoring (including data quality, data completeness, system usability, and system acceptability). We will attempt to explore disease trajectory, patient stratification, and identification of acute clinical events such as exacerbations. A key aspect is understanding the potential of real-time data collection. We will simulate an intervention to acquire responses at the time of the event to assess model performance for exacerbation identification.

**Results:**

The Remote Assessment of Lung Disease and Impact on Physical and Mental Health (RALPMH) study provides a unique opportunity to assess the use of remote monitoring in the evaluation of lung disorders. The study started in the middle of June 2021. The data collection apparatus, questionnaires, and wearable integrations were setup and tested by the clinical teams prior to the start of recruitment. While recruitment is ongoing, real-time exacerbation identification models are currently being constructed. The models will be pretrained daily on data of previous days, but the inference will be run in real time.

**Conclusions:**

The RALPMH study will provide a reference infrastructure for remote monitoring of lung diseases. It specifically involves information regarding the feasibility and acceptability of remote monitoring and the potential of real-time data collection and analysis in the context of chronic lung disorders. It will help plan and inform decisions in future studies in the area of respiratory health.

**Trial Registration:**

ISRCTN Registry ISRCTN16275601; https://www.isrctn.com/ISRCTN16275601

**International Registered Report Identifier (IRRID):**

PRR1-10.2196/28873

## Introduction

### Background

Patients with chronic conditions like chronic obstructive pulmonary disease (COPD) and interstitial lung disease (ILD) must often manage their diseases from a community setting, and this presents natural challenges in monitoring patient health status. Currently, COVID-19 presents additional challenges, especially for vulnerable patients with pre-existing conditions and diseases where, due to shielding, their routine care cannot be performed properly [1]. Remote monitoring of the physiology and symptoms of patients via wearable devices could provide convenient and useful advantages over conventional care for patients managing their health care in real-world settings. These can include detailed information on their historical health, current health status, and potential to intervene during acute events, as well as prognosis of future health and disease trajectory. Remote monitoring may also provide an opportunity during events like the COVID-19 pandemic to safely monitor disease exacerbation or progression without putting patients in situations where risk of exposure to COVID-19 is increased.

### Remote Monitoring

This study aims to use the open-source RADAR-Base (Remote Assessment of Disease and Relapse) mobile health (mHealth) platform to collect and analyze multiple data sets associated with respiratory disorders. Several cardiopulmonary parameters are now available in modern consumer wearable devices, and due to close coupling of the heart and lungs, measurements of the functions of these organs are expected to provide good characterization of diseases. This study will include continuous data collected from wearable devices (eg, heart rate [HR], respiratory rate, and oxygen saturation [SpO_2_]), including pulse oximeters and spirometers, mobile phones (audio), digital tests, and smartphone symptom questionnaires.

The RADAR-Base community emerged from the Innovative Medicines Initiative (IMI) project RADAR-CNS (Remote Assessment of Disease and Relapse – Central Nervous System), where a consortium of clinicians, developers, researchers, patient organizations, and European Federation of Pharmaceutical Industries and Associations (EFPIA) partners joined forces to explore the potential use of sensor data from wearable devices like fitness trackers and smartphones in research and health care. The RADAR-Base platform is a scalable and interoperable mHealth platform that provides capabilities for remote monitoring passively (eg, sensor data, wearables, and internet of things [IoT]) and actively (eg, questionnaires and digital tests). The platform developed at King’s College London and the Hyve in the Netherlands is already being used in a number of large-scale longitudinal mental and physical health–related disorder projects [2,3]. The complete RADAR-Base technology stack is available under an Apache 2 open-source license and is supported by an active community of developers, researchers, and clinicians who focus on continuously improving data quality, user experience, and validation, and extending the platform with new features and data sources.

All the data collected and aggregated using the RADAR-Base platform are standardized using Avro schemas [4] and harmonized across various data streams.

RADAR-Base also provides the potential to respond or alert in near real time based on some state of the data being collected; this could include identifying, for example, an exacerbation and triggering a response, such as an intervention or follow-up questionnaire/test or confirmation.

This pilot will help answer how remote monitoring may be used for lung disease patients, who in many cases may be shielding during the COVID-19 pandemic because of being at high risk and being vulnerable, and offers additional benefits, including participation without additional risk of travel or interaction with hospital staff.

### ILD

ILD, or lung fibrosis, is one of a spectrum of fibrotic diseases associated with aging, obesity, diabetes, and pollution, which are responsible for approximately 45% (9/20) of premature deaths in Western Europe. Of over 90,000 patients in the United Kingdom with ILD, approximately 30,000 have idiopathic pulmonary fibrosis (IPF), which is the most severe form. IPF is a disease of unknown etiology that is more frequent in males and presents mainly in the sixth and seventh decades of life [5]. There is no cure, and the median survival of 3 to 5 years following diagnosis is worse than that for many cancers. As the fibrosis progresses, there is impaired pulmonary function, respiratory failure, and ultimately death. Throughout its course, IPF has significant effects on physical (dyspnea, dry cough, weight loss, and fatigue) and social (recreational activities and relationships) functions, with severe consequences for the patient’s health-related quality of life (QoL). Clinical courses are punctuated by episodes of worsening disease that may result in death. These acute exacerbations of IPF (AE-IPF) are estimated to occur in 4% to 20% (1-4 in 20) of patients each year, but the true incidence and impact are not known [6].

Management of AE-IPF involves establishing the diagnosis and excluding other causes of increasing dyspnea, excluding infection, and considering the use of steroids, antibiotics, and/or anticoagulation, none of which has been shown to be of benefit. The trajectory of patients with IPF is heterogeneous with great variability in the disease course. Some progress slowly, whereas others progress more rapidly, and this can cause emotional distress and anxiety. Patient-reported outcome measures are used to measure health-related QoL, assess symptoms, and evaluate disease progression. The management of patients with IPF is multifaceted and consists of patient education and support, regular outpatient surveillance, symptom relief, pulmonary rehabilitation, annual vaccinations to prevent respiratory infection, identification and management of AE-IPF, supplemental oxygen, management of comorbidities, and ultimately palliative care or, in a minority of patients, referral for lung transplantation [7].

Two antifibrotic treatments (pirfenidone and nintedanib) are available for patients who meet the stringent National Institute for Health and Care Excellence (NICE) criteria. They neither cure nor reverse the fibrosis and have little impact on symptoms, but they have been shown to reduce the rate of lung function decline, and pirfenidone has been shown to reduce AE-IPF and improve progression-free survival [8].

With regard to the impact on health care systems, IPF is a cost- and resource-intensive disease encompassing hospitalization, home care, long-term care, and antifibrotic therapy. The full health burden on the National Health Service (NHS) and UK economy is unknown, but data from the British Lung Foundation and projected estimates from our patient cohort suggest that there are approximately 30,000 IPF diagnoses each year. Health care costs alone for IPF are estimated at US $15,000 to $78,000 per patient-year [6].

Regarding the need for biomarkers for precision management, the disease progression in IPF is highly variable with individuals experiencing very different trajectories. Response to antifibrotic therapy is also inconsistent with some patients tolerating the medication well and others experiencing significant side effects. Currently, there is a lack of valid endpoints, apart from a change in forced vital capacity, which has poor sensitivity and specificity to accurately assess disease activity or response to treatment [9]. This makes it difficult to predict individual prognosis or reliably detect early treatment response or failure, which is important for developing treatment plans and providing patients with accurate prognostic information, which allows them to plan for their future. Remote monitoring may allow clinicians and patients access to more granular longitudinal data on disease progression, the rate of AE-IPF, and effects on QoL, and begin to offer personalized treatment approaches in this cohort. Remote monitoring may also reduce patients’ attendance at the hospital for clinical follow-up or when taking part in clinical trials of novel agents. Remote monitoring may allow the early identification of AE and a better understanding of the frequency and impact of these events, and may provide the potential to develop clinical trials of treatments in these patient groups.

### COPD

COPD is a common long-term condition of the lungs that is usually caused by cigarette smoking. In addition to daily symptoms and limitations in activities, patients are prone to developing chest infections called “exacerbations” [10,11]. Exacerbations are a significant problem. They are unpleasant for patients and sometimes severe enough to cause hospital admission (and therefore NHS pressures) and death. Reducing the impact of exacerbations is very important [12]. We have previously shown that earlier treatment of COPD exacerbations results in faster recovery and reduced chance of hospital admission. Helping patients to detect exacerbations early is therefore important. We have also recently shown that monitoring HR and oxygen saturation via a finger probe may assist in this, especially overnight when the physiological signal is cleaner [13]. Integration of these signals with additional symptom data and use of innovative data analysis methodology are likely to result in the greatest chance of supporting the early detection of exacerbations and assessment of disease progression. This is even more important in the era of COVID where many patients with COPD are classified as “clinically extremely vulnerable,” and thus, remote monitoring provides the safest way to support management in partnership with their clinicians.

### Posthospitalization COVID-19 Lung Disorders

Recovery from COVID-19 has many unknowns, especially in the long term [14]. The symptoms of COVID-19 have varied among those who have tested positive. Some have displayed no symptoms, while others have developed severe pneumonia, progressing to lung injury, and acute respiratory distress syndrome, as well as pulmonary fibrosis in the longer term. Notably, the consequences of COVID-19 include effects on other organs, including the heart, kidneys, and brain. Correspondingly, a diverse set of associations have been observed that together have been called “long COVID,” which involves prolonged and delayed recovery from the acute illness, including fatigue, shortness of breath, and cough, associated with mental health and neurological disorders, such as fatigue, trauma, and anxiety/depression [15,16]. For those who were hospitalized and have since been discharged, it is not yet clear what their medical, psychological, and rehabilitation needs will be to enable them to make as full a recovery as possible.

Given this need to follow-up with COVID-19 patients after hospitalization, we consider remote monitoring to provide some key opportunities. First, observation of chronic symptoms will necessitate home-based monitoring, as the scope of regularly interfacing with participants in the clinic may be limited due to the likelihood of further periods of lockdown and self-isolation of this population. Second, there needs to be a greater focus on understanding how daily life is affected by this disease. Remote monitoring, therefore, provides an ideal opportunity to collect multiple continuous data streams from participants to report on physiology, QoL, and environmental and functional aspects. Building on our existing experience in using wearables to monitor participants who develop COVID-19 [17], we aim to extend this to enable detailed observation of patients as they experience symptoms of long COVID. By using a longitudinal, high frequency, and largely passive monitoring approach, we aim to develop an understanding of the disease trajectory and the fluctuation of symptoms.

### Theoretical Framework

The Remote Assessment of Lung Disease and Impact on Physical and Mental Health (RALPMH) study will use a prospective cohort study framework. This will leverage our RADAR-Base software platform and existing experience working on remote monitoring projects, such as RADAR-CNS [18], and take a similar approach to the major depressive disorder protocol [19]. In addition to this, we have recently developed RADAR-Base capabilities to deliver notifications dependent on real-time processing of participant data streams. This module of RADAR-Base will be evaluated here by deploying an exacerbation detection algorithm (using HR, SpO_2_, and other measures), and participants will be asked in near real time to confirm/reject and score the algorithm’s assertion via a short questionnaire, the Exacerbation Rating Scale (ERS), close to or during the period of exacerbation, in order to provide accurate feedback independent of recall. The ERS scoring will be used to evaluate the algorithm sensitivity/specificity and to evaluate options for personalized exacerbation detection.

**Figure 1:**
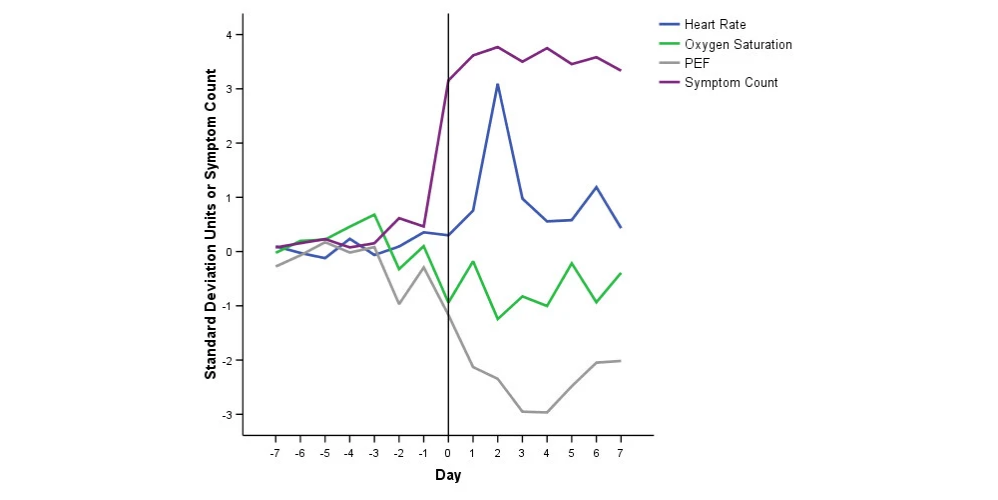
Time course of symptoms and oximetry variables at exacerbation [11]. PEF: peak airflow.

Previous studies [12,13] on exacerbations in COPD found that changes in resting heart rate (RHR), pulse oxygen saturation (SpO_2_), and peak airflow are highly correlated with symptoms before, during, and after exacerbation onset, as shown in Figure 1.

Another study [20] looked at the identification and subsequent prediction of exacerbations in COPD. The authors used features derived from pulse oximeters to predict exacerbations using logistic regression. They found that all three vital signs (oxygen saturation, pulse rate, and respiratory rate) are predictors of exacerbations, with oxygen saturation being the most predictive. Another study [21], which looked at the correlation between RHR and acute exacerbations in COPD, found that patients with a higher RHR following exacerbation demonstrated an increased risk of exacerbation.

There is evidence in the literature that pulmonary diseases, like COPD, lung fibrosis, etc, are closely related to the heart [22]. Pulmonary vascular abnormalities are frequently present in patients with respiratory disorders. Similar correlations were found with HR in severe acute respiratory syndrome (SARS), where patients were found to have a high HR and low blood pressure [23].

One interesting and relatively novel approach to understanding the changes in respiratory disorders is through capturing breathing sounds to measure the breathing rate and detect features such as wheezing, coughing, sneezing, and snoring, using audio data. Two exciting works in this field are mLung++ [24] and SonarBeat [25].

The mapping between data types and analytical approaches under consideration is shown in Table 1.

**Table 1:** Data analysis methods.

Data type	Methods	Labels
Active raw audio	MFCC^a^, SVM^b^, Adaboost	Questionnaires, tasks, spirometry
Passive wearable sensor	KNN^c^, least squares regression, Adaboost, HMM^d^	Questionnaires, tasks, event diary, spirometry
Multimodal sensor	DeepSense (CNN^e^+RNN^f^), GIR^g^, hierarchical HMM	Questionnaires, tasks, spirometry

^a^MFCC: mel-frequency cepstral coefficients.

^b^SVM: support vector machine.

^c^KNN: K-nearest neighbor.

^d^HMM: hidden Markov model.

^e^CNN: convolutional neural network.

^f^RNN: recurrent neural network.

^g^GIR: global iterative replacement.

### Aims

Our goal is to investigate the acceptability and feasibility of remote monitoring of patients with pulmonary disorders for the quantification of symptoms, understanding of the disease trajectory, and detection and prediction of clinically important events, such as exacerbations, in the following three disorder areas: COPD, ILD, posthospitalization COVID.

### Objectives

#### Primary Objective

The primary aim of the study is to evaluate cardiopulmonary disorders as potential targets for real-time, continuous, real-world remote monitoring. This study will investigate the potential benefit, acceptability, and feasibility of multiparametric remote monitoring of patient symptoms and physiology using commercially available wearable sensors for HR, activity, and SpO_2_; spirometry; phone sensors; questionnaires; and digital tests in patients with a range of pulmonary disorders.

The evaluation will be based on patient acceptability, dropout rates, and interpretation of data; detection of clinically important events, such as exacerbations and disease progression; quantification of symptoms (physical and mental health); impact of the disease on mood and well-being/QoL; and trajectory tracking of main outcome variables, symptom fluctuations, and order.

#### Secondary Objective

The secondary objective of this study is to provide data for power calculations [26] for a follow-on study. Power calculations will be centered around understanding the number of exacerbations according to sample size and duration. The power is effectively how good the signal is.

This will help plan future studies where we need to decide the sample size and duration to obtain accurate and acceptable exacerbations and the data associated with them.

### Outcomes

#### Acceptability of the Remote Monitoring System in the Three Disease Areas

The acceptability of the platform will be determined in terms of recruitment, retention, data completeness, and the qualitative experience of participants. The exit survey Technology Assessment Model Fast Form (TAM-FF) will also be used to evaluate the data collection infrastructure with participant feedback. The study will test both the feasibility and acceptability of tasks for participants. On completion of the data collection period, we will measure the total available data as a function of a theoretical maximum and assess data quality measured by a range of criteria, including missingness and contiguity.

#### Assess the Potential of Remote Monitoring in COPD, IPF, and COVID-19

The potential of remote monitoring will be evaluated in the context of cardiopulmonary disorders. This will involve developing methods to quantify disease trajectory as compared with standard clinical measures (in IPF, these would be changes in forced vital capacity and death); detecting exacerbations/symptoms, such as changes in wearable data (eg, HR, SpO_2_, and activity), during the reported period of exacerbation (a real-time algorithm will be included to predict exacerbations with patients notified with the ERS to confirm the prediction at or close to the time of the event); detecting exacerbations prior to or after the reported period of exacerbation (eg, a signal that may precede participant awareness of the exacerbation/symptom); detecting subclinical exacerbations in patients with lung fibrosis; tracking self-reported symptoms and outcomes (including precursor presymptomatic signals) and their frequency and order; and reporting longitudinal mental health symptom measures, as reported by the Generalized Anxiety Disorder scale (GAD-7) and Patient Health Questionnaire depression scale (PHQ-8), associated with the three diseases. This will provide the potential to assist with future applications around disease self-management. In the case of the posthospitalization COVID cohort, we will assess whether remote monitoring can be used as a symptom collection tool and as a long-term low-burden monitoring solution. Fatigue will be assessed by Garmin Body Battery values and the Fatigue Severity Scale, while long-term COVID impairments will be measured weekly using the Centers for Disease Control and Prevention (CDC) COVID-19 long-term effects (CCLTE) list, World Health Organization (WHO) symptoms list, and post-COVID functional status (PCFS), a COVID-19-specific widely used questionnaire on health-related impairments (physical functioning scale).

#### Data for Future Calculations

This study will provide data and information for future power calculations for larger cohort studies, including informative data types established by analysis of correlates with symptoms or outcomes of interest. An informative minimal data set can then be derived from this superset. This will also provide the unit cost of data collection for the full and minimal data sets for the planning of any future studies.

Remote monitoring provides the opportunity to continuously monitor patients in their daily lives outside of the hospital, with the potential to automate the detection of disease exacerbations and monitor the long-term evolution of disease trajectory. The acceptability and feasibility of remote monitoring using measures of heart and lung functions in patients with lung diseases are necessary first steps in this process, which this study aims to evaluate.

## Methods

### Study Design: Methods of Data Collection

#### RADAR-Base mHealth Platform

##### Active Data Collection

**Figure 2:**
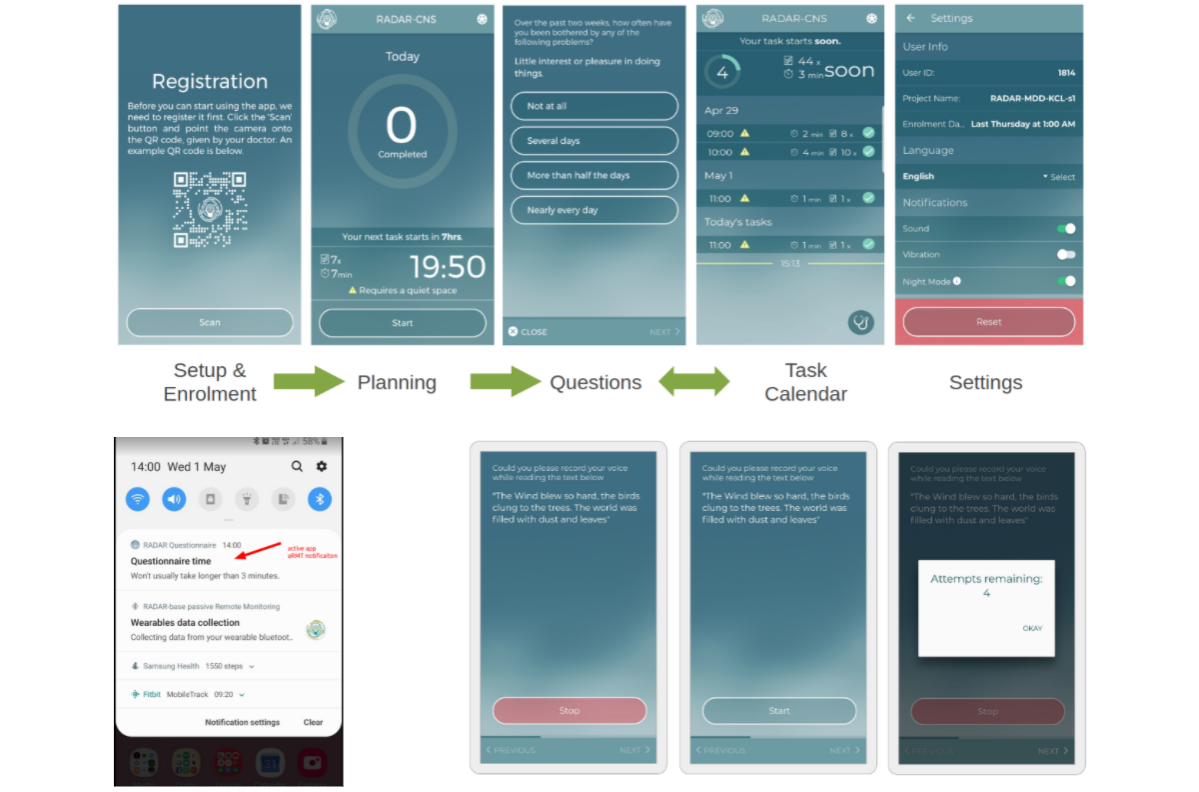
The Active Remote Monitoring Technology (aRMT) app used to collect phone-delivered questionnaires. Top: app screens and example questionnaire. Bottom left: Example notification to complete the questionnaire. Bottom right: Speech task.

The Active Remote Monitoring Technology (aRMT) app (Android and iOS) will be used to collect data from patients by issuing questionnaires and tests that require some conscious action to perform. These will include questionnaires for participant QoL and mental health (GAD-7 and PHQ-8) and disorder-specific questionnaires for symptom tracking of COPD (COPD Assessment Test), ILD (Living with Idiopathic Pulmonary Fibrosis assessment), and posthospitalization COVID (PCFS, CCLTE list, and WHO COVID-19 symptoms list). These are summarized in Table 2. Participants will be issued a notification (at the appropriate time) that will open the corresponding questionnaire to be filled on the mobile app. Further to the scheduled questionnaires, the app will be used to generate dynamic notifications for questionnaires to validate the performance of symptom classification and prediction in near real time. Details of the aRMT mobile app are provided in Figure 2.

This study will also include a battery of experimental digital tests to explore the potential to assess lung breathing function through the use of audio capture or other interactive means. Audio data are readily available through the phone’s built-in microphone (or the addition of an auxiliary Bluetooth microphone for improved or standardized sound capture). Participants will be issued a notification to complete the relevant task, and selecting this will open the corresponding test on the mobile phone with instructions on how the test is to be performed. Active audio tasks, such as pronouncing sustained vowels and counting from 1 to 20, will provide additional information on voice production dynamics that might be affected by lung disorder symptoms. A lung sound test, which will record audio during breathing by placing the microphone against the chest during a sequence of breaths [42,43], might be evaluated in a further study based on this protocol.

Furthermore, the audio tasks will be validated in conjunction with patient tests and protocol development to ensure that they capture relevant information. Quality assurance mechanisms will be implemented to ensure that incoming audio signals are valid (eg, checking if the signal-to-noise ratio is within an acceptable scope or if the voice is contained within a sample).

**Table 2:** Remote monitoring measures.

Remote monitoring parameter		Data source	Collection frequency	Cohort	Purpose
**Speech and audio**					
	Speech – Active Remote Monitoring Technology (aRMT)	Digital test/aRMT phone app	Weekly	All	Voice production tasks via the phone. These tasks will assess change in the phonatory-respiratory system [3].
**Activity, functioning, and fatigue**					
	Activity	Wrist wearable device	Continuous	All	Measure exercise levels, and combine and compare heart rate (HR) for the measurement of proportional nonresting HR. Impacts on lifestyle, including physical activity and mobility [27].
	Sleep parameters	Wrist wearable device	Continuous	All	Evaluation of the duration and quality of sleep [28].
	Fatigue severity scale	Questionnaire/aRMT phone app	Weekly	All	Subjective experience of fatigue [29].
	Passive fatigue measure	Wrist wearable device	Continuous	All	Heart rate variability (HRV), time to bed, and Garmin Body Battery level [30,31].
**Cardiopulmonary**					
	HR	Wrist wearable device	Continuous	All	Continuous measure of baseline HR for (1) resting HR (sedentary and sleeping), (2) nonresting HR (under light, medium, and high activity or stair climbs), and (3) cardiopulmonary performance [32].
	HRV	Wrist wearable device	Continuous	All	Continuous measure of the variation in time intervals between consecutive heartbeats. Low resting HRV is an indication of high levels of physical or mental stress [33].
	Respiratory rate	Wrist wearable device	Continuous	All	Respiration rate [34].
	Pulse oximeter (SpO_2_)	Wrist wearable device (continuous)	Continuous (nighttime)	All	Blood oxygenation as measured by PhotoPlethysmoGram sensors on the wrist wearable device; this measurement should be continuous at least during the nighttime [35].
	Pulse oximeter (SpO_2_)	Finger pulse oximeter (periodic)	Daily	All	Periodic assessment with a clinically approved finger-worn device will be provided to validate the daily measure and may be included for dynamic spot checks.
	Breathing	Digital test/aRMT phone app	Weekly	All	Measure lung function and volume by inspiration and expiration using tests delivered through the aRMT app and audio capture.
	Spirometry	Spirometer	Daily	All	Lung function measurement.
**Questionnaires - Symptoms, mental health, quality of life (QoL) (aRMT)**					
	Post-COVID-19 functional status (PCFS) scale	Questionnaire/aRMT phone app	Weekly	COVID-19	Establish post COVID-19 functional status [36].
	CDC COVID-19 long-term effects (CCLTE) (aRMT)	Questionnaire/aRMT phone app	Daily	COVID-19	Establish the degree of long-term COVID-19 effects [15].
	WHO COVID-19 symptoms (WCS) list (aRMT)	Questionnaire/aRMT phone app	Daily	COVID-19	Establish the degree of persistent COVID-19 symptoms [37].
	COPD assessment test (CAT)	Questionnaire/aRMT phone app	Cohort: Daily	All	CAT to measure the impact of COPD on a person’s life. It is unidimensional, and assesses cough, sputum, dyspnea, and chest tightness. Eight questions [38,39].
	Idiopathic pulmonary fibrosis patient-reported outcome measure	Questionnaire/aRMT phone app	Weekly	Interstitial lung disease (ILD)	ILD QoL self-report.
	Living with idiopathic pulmonary fibrosis	Questionnaire/aRMT phone app	Daily	ILD	ILD QoL self-report.
	Visual analog scale (VAS) cough	Questionnaire/aRMT phone app	Monthly	ILD	Score symptoms (cough).
	St. George’s Respiratory Questionnaire (SGRQ)	Questionnaire/aRMT phone app	Quarterly	All	Assess the impact of overall health, daily life, and well-being in patients.
	Pittsburgh Sleep Quality Index (PSQI)	Questionnaire/aRMT phone app	Monthly	ILD	Sleep scoring questionnaire.
	Exacerbation rating scale (ERS)	Questionnaire/aRMT phone app	Dynamic/on demand	All	A confirmatory rating scale for detected exacerbations in real time; participants will be sent notifications to complete these.
	Generalized Anxiety Disorder scale (GAD-7) and Patient Health Questionnaire depression scale (PHQ-8) (aRMT)	Questionnaire/aRMT phone app	Fortnightly	All	Establish depressive and anxiety symptoms [40,41].
**Electronic case report form (eCRF) and surveys**					
	Epworth sleepiness scale	eCRF REDCap	Baseline	All	Used to diagnose obstructive sleep apnea (OSA).
	STOPBang questionnaire	eCRF REDCap	Baseline	All	Used to diagnose OSA.
	MRC breathlessness	eCRF REDCap	Baseline	All	Dyspnea scale that is used to evaluate the impact of breathlessness on daily activity.
	Demographics	eCRF REDCap	Baseline	All	Patient demographics form.
	Study information	eCRF REDCap	Baseline	All	Study-related information collected at baseline, for example, phone and device registration, and administrative information.
	Contact information	Local Site File	Baseline	All	Contact information.
	Technology Assessment Measurement Fast Form	eCRF REDCap	End of study	All	Measure the impact of the technology being used and evaluate its acceptability, usability, and performance.
	Experience of participation	eCRF REDCap	End of study	All	Exit interview (semistructured).

##### Passive/Background Data Collection

Garmin Vivoactive 4 is the selected wrist-worn wearable device. Several parameters of interest are reported continuously by the wearable device, including activity (steps and exercise as calories consumed), sleep, fatigue, HR, fatigue levels (Garmin Body Battery), HR variability, respiratory rate (RR), and pulse oximetry (SpO_2_) (Table 2).

Additional periodic data collection will be done with finger pulse oximetry and spirometry (Table 2). Participants will receive a notification to carry out pulse oximeter and spirometry tests at a convenient time on a daily basis, and values will be recorded on the aRMT app.

#### Data Sources

Data sources used in this study include wearable devices, mobile phone apps, and questionnaires. All the measures collected are shown in Table 2.

#### Clinical Data

Participants will be requested to consent to their medical records (routine clinical and GP records; via the clinical team) and anonymized data being made available to the study team throughout the study. Where possible, specific data sets from routinely acquired clinical records may be included.

### Data Analysis

Multimedia Appendix 1 summarizes the data analysis algorithms and models under consideration in this study.

Analyses are intended to assess the feasibility of the RADAR-Base platform for lung disorder remote monitoring (including the quality of data, the cross-section of passive and active data, data completeness, the usability of the system, and the acceptability of the system).

We will generate descriptive statistics for demographics, attrition rate, and the number of participants using the remote assessment measurements without loss or damage and providing adequate quantity and quality of data, and for the duration of the study period.

Using classification/regression/machine learning approaches, we will investigate whether any demographics and/or other numerical information obtained during the baseline and longitudinal data collection periods of the study might serve as predictors for subject dropout and the percentage of adequate data.

We will establish the appropriate setup of data collection and the parameters of the study, which would be required to conduct a future larger longitudinal study.

Feasibility and acceptability of the wearable device will be assessed by answering the following questions: (1) Are the sensors on the device (not a gold standard) feasible and acceptable to conduct remote monitoring of pulmonary diseases? (2) Is Garmin a feasible device for measuring changes in key physiological parameters such as HR and SpO_2_? These will be evaluated against the gold standard device (pulse oximeter and spirometry).

The feasibility of the symptom questionnaire will be evaluated against the gold standard from spirometry. Technology Assessment Measurement Fast Form (TAMFF) plus the experience of participation exit interviews will be used to determine the overall feasibility and acceptability of the technology and the protocol used in the study.

If adequate data are collected in the pilot, we aim to explore methods to establish the feasibility of the data collection apparatus as a means to study disease trajectory and patient stratification.

This will involve using data involving HR, SpO_2_, sleep, activity, respiratory rate, questionnaires, and other collected measures to model participant stratification and differential disease trajectory.

We will explore the identification of acute clinically interesting events, such as exacerbations and rapid changes in clinical conditions or physiological data streams.

We will use the pulse-respiration quotient [44] and pulse-activity quotient as measures of the changes in respiration efficacy and exacerbation.

We will adopt machine learning approaches to perform both cross-sectional and individualized classification for the identification of events, such as exacerbations, using a questionnaire or other active data as labels providing context to passive data streams such as HR, SpO_2_, respiratory rate, and others.

Using multimodal data sets, we will characterize the periods of time around acute events, such as exacerbations, to include the pre-exacerbation period, period during exacerbation, and postexacerbation period.

We will investigate the potential of the data collected to identify putative subclinical exacerbations or other lower level fluctuations in participant symptoms.

If adequate numbers of events are generated in the study, an opportunity to apply anomaly/novelty detection methods will be possible. In this way, we will use these approaches to learn the baseline state for the participants and establish significant deviations from this.

Using the real-time aspect of the data collection, we will use the real-time ERS to acquire real-time responses to evaluate and assess the performance of a model for exacerbation detection and refine an individual-level model for exacerbation detection.

Since we do not have enough prior data, we plan to consider real-time anomaly detection methods and preprocess the data based on prior knowledge. The models will be pretrained on a daily schedule (using previous N days of data; N is yet to be decided but is initially considered 21 days) and will be ready for running inferences in real time (using wearable sensor data as inputs collected in real time) and taking actions based on the results of the inferences (sending an ERS assessment through the aRMT app) to acquire responses at the time of the event to assess the performance of the model.

### Study Setting

The main study setting will be remote and will involve near real-time home-based monitoring, and data will principally be collected under this setting. Participants will also attend baseline and exit face-to-face visits with the clinical team, during which initial baseline and exit data will be obtained and assessments will be conducted.

### Sample and Recruitment

#### Inclusion Criteria

The inclusion criteria are presented in Table 3.

**Table 3:** Inclusion criteria.

Criteria	COPD^a^	ILD^b^	PH-COVID^c^
Clinical conditions	20 patients with a diagnosis of COPD	20 patients with a diagnosis of ILD	A clinical diagnosis of COVID-19 (within 4-13 weeks of enrollment) who report symptoms interfering with day-to-day activity present for more than 28 days following the onset of COVID-19
Gender	Male/Female	Male/Female	Male/Female
Age range (years)	18+	18-90	18+
Prior mobile phone use	Required	Required	Required
Willingness to use monitoring devices and complete study questionnaires	Required	Required	Required
History of exacerbation	2 or more exacerbations in the last 1 year	N/A^d^	N/A

^a^COPD: chronic obstructive pulmonary disease.

^b^ILD: interstitial lung disease.

^c^PH-COVID: posthospitalization COVID.

^d^N/A: not applicable.

#### Exclusion Criteria

The exclusion criteria for all groups (COPD, ILD, and posthospitalization COVID) are as follows: non-English language speaker, lack of physical capability to participate (eg, heart failure), pregnancy, and lack of capability to consent.

### Sampling

Convenient sampling will be employed for this pilot study.

#### Sample Size

The study will include 20 participants for each of the three disease areas. This is a small sample feasibility study to assess the practical use of remote monitoring in three lung disease areas. The sample size will adequately allow objective assessment of the system deployed for this type of data collection in the typical patient population.

#### Sampling Technique

Sequential participants who fit the inclusion/exclusion criteria will be identified from the respiratory outpatient clinics at the University College London Hospital (UCLH) and Royal Free Hospital (RFH).

### Recruitment

Participants who fit the inclusion/exclusion criteria will be identified from the respiratory outpatient clinics at the UCLH and RFH.

### Sample Identification

#### Participant Search and Consent

Recruitment will be done either in person (via a cleanroom) or remotely (by phone or video call) by clinicians, who will provide information about the study in an easily accessible form reviewed by the patient advisory board. As part of the consent process, participants will be informed that the data they provide will not be actionable (in other words, it will not trigger a clinical investigation or intervention). Written informed consent will be obtained prior to performing any study assessments or procedures. Participants will be requested to consent to their medical records being made available to the study team throughout the study.

#### Participant Cohorts

##### ILD/IPF

The ILD/IPF cohort will include 20 participants recruited from the UCLH ILD service followed for 6 months. We will recruit patients across the spectrum of progressive to stable disease. We will analyze whether monitoring is able to detect progression earlier than the current standard (lung function test performed every 3 months or patient reporting to a clinician) and could help us identify progression earlier.

##### COPD

We will recruit 20 patients with COPD from our services in London and follow them for up to 6 months or until the first exacerbation, whichever is sooner. We will recruit patients with a history of exacerbations to increase the likelihood of identifying patients who would experience events during the study. We will analyze whether monitoring is able to detect exacerbations earlier than the current gold standard (patient reporting to a clinician) and therefore could be used to help patients get treatment earlier.

##### Posthospitalization COVID

For UCLH participants, we will define the population under study as people with a clinical diagnosis of COVID-19 (within 4-13 weeks of enrollment) and who report troublesome symptoms interfering with day-to-day activity present for more than 28 days following the onset of COVID-19 (n=20). A baseline assessment will be conducted remotely via a web-based questionnaire, which will obtain information on demographics, medical and psychiatric history, health behaviors, and medications. Participants will be asked about the symptoms, severity, and consequences (eg, hospitalization and ventilatory support) of their acute illness.

### Consent

Informed consent will be sought at the end of the screening process and prior to the baseline clinical team meeting. Patients will have the study explained to them and will be given a copy of the participant information sheet (PIS). They will be given adequate time to consider taking part in the study and to ask any questions. Patients who are unable to give informed consent will not be recruited. Due to the nature of the remote monitoring study, we will optionally include a remote consent process for participants who are not able to attend the clinic in person. The remote consent process will be implemented using the REDCap electronic case report form (eCRF) e-Consent module to deliver the consent form; however, it is our preference to seek to keep wet signature/paper-based consent forms. These will be held both as electronic copies and hard copies.

Upon receipt of consent forms, participants will be booked for the enrollment training session. Prior to the session, participants will be provided a RALPMH study pack including study devices (Garmin, spirometer, and finger pulse oximeter) and equipment either by post or during a face-to-face session (via a cleanroom) if possible. Participants will receive a 45- to 60-minute training session on the use of the wearable devices, mobile sensors, and aRMT smartphone app. This will include a PIS leaflet summarizing key information and researcher contact details for future reference. The purposes of the study will be clearly explained, and participants will be provided with practical information, such as how to switch devices on and off, how to charge devices, and how to respond to questionnaires and digital tests on a notification via the app. Participants will receive a follow-up call 1 week after the start of data recording for any additional support as required. If participants do not have a suitable phone, we may provide one from a limited number of reserve devices. Precreated accounts for devices (eg, Garmin) will be registered using a study email address (eg, RALPMH022@domain.com) and dummy contact details. Baseline data will be collected in this first session via the study REDCap eCRF project instruments.

## Results

### Study Flow Chart

**Figure 3:**
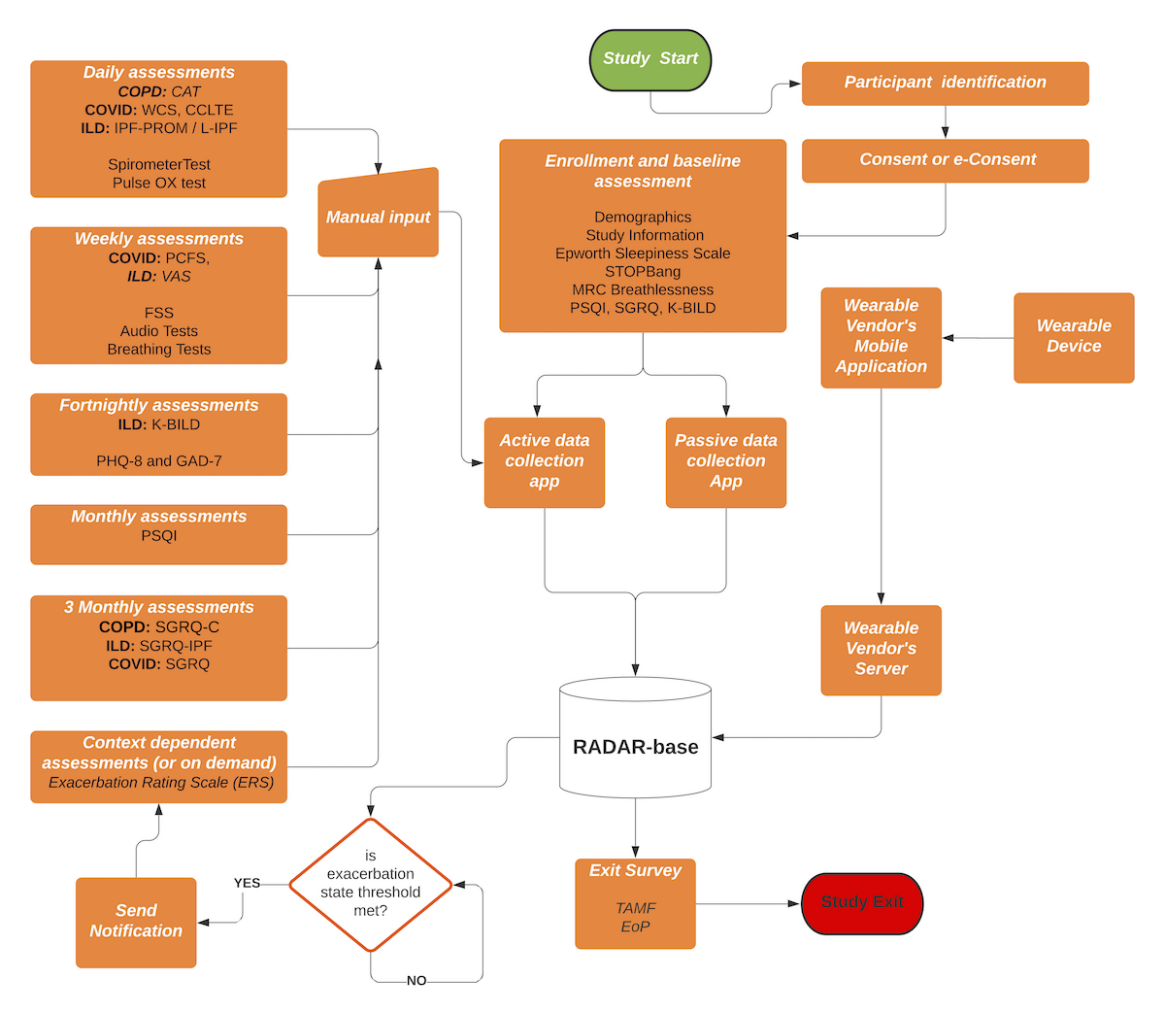
Study flow diagram. CAT: COPD assessment test; CCLTE: CDC COVID-19 long-term effects; COPD: chronic obstructive pulmonary disease; EoP: experience of participation; ERS: Exacerbation Rating Scale; FSS: Fatigue Severity Scale; GAD-7: Generalized Anxiety Disorder scale; ILD: interstitial lung disease; IPF: idiopathic pulmonary fibrosis; l-IPF: living with idiopathic pulmonary fibrosis; KBILD: The King's Brief Interstitial Lung Disease; MRC: Medical Research Council; PCFS: post-COVID function status; PHQ-8: Patient Health Questionnaire depression scale; PROM: patient-reported outcome measure; PSQI: Pittsburgh Sleep Quality Index; RADAR: Remote Assessment of Disease and Relapse; SGRQ: St. George's Respiratory Questionnaire; TAMF: Technology Acceptance Model Fast Form; VAS: visual analog scale; WCS: World Health Organization COVID-19 symptoms.

The study flowchart is shown in Figure 3. At the start of the study, participants remotely consent and are provided with the PIS. They are then provided a set of enrollment or baseline assessments to complete online.

All the scheduled active assessments (self-reports) that need manual input periodically are shown on the left, which are piped through the active data collection app to the RADAR-Base platform.

The wearable device provided to participants connects to the wearable vendor’s application, and the data are uploaded to the vendor’s server, which is then synchronized to the RADAR-Base platform. On getting all the data, the RADAR-Base platform runs real-time data processing, and based on a threshold for exacerbation, it sends a notification and assessment for confirmation of the exacerbation in the form of the ERS. On exit or completion of the study, the participants will be asked about their experiences with the technology and the study.

### Timeline

**Figure 4:**
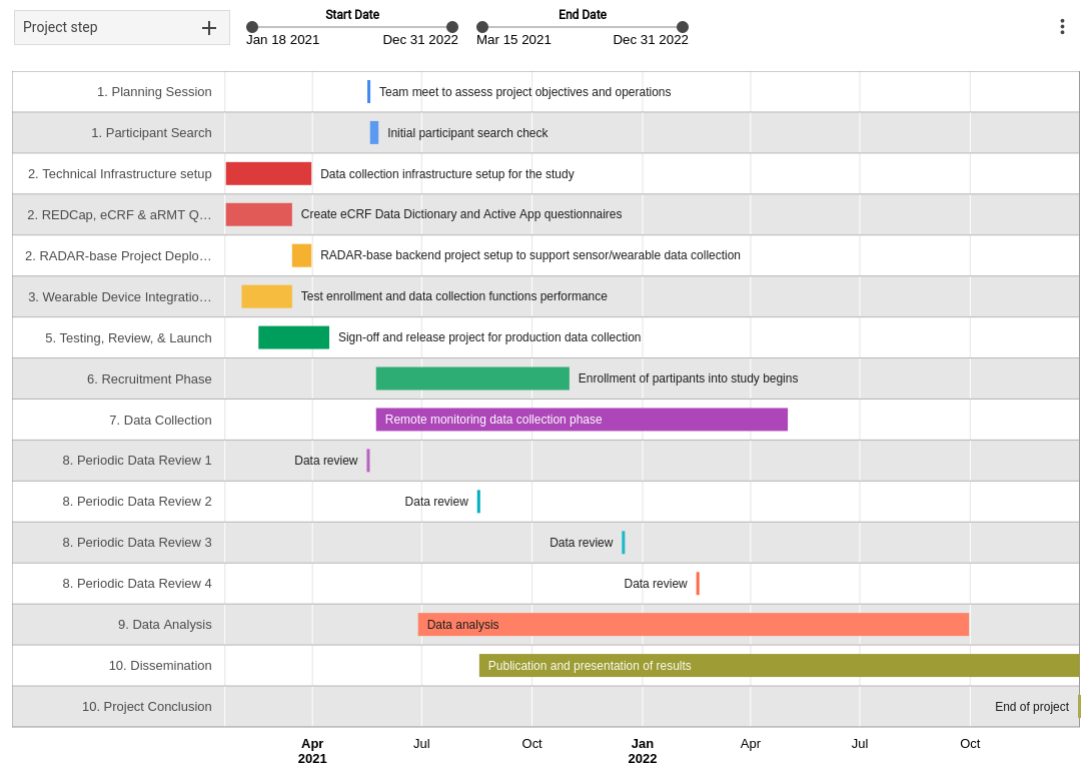
Study timeline. aRMT: Active Remote Monitoring Technology; eCRF: electronic case report form; RADAR: Remote Assessment of Disease and Relapse.

The timeline for the study is shown in Figure 4. The planning and technical support for the study were setup in March 2021. The recruitment and data collection phase has started in June 2021 and will end in June 2022 (1 year). At various points during this period, a review of the data quality and quantity will be performed. Data processing and analysis has started in July 2021 and is planned to end in October 2022. The publication writeup and final publication are planned from September 2021 to December 2022.

### Progress to Date

The study started recruiting in the middle of June 2021. The data collection apparatus was setup and tested by clinical teams in April 24, 2021. All the questionnaires and their schedules are being served either via the RADAR active application or REDCap. Device selection (oximeter, spirometer, and wearable) was completed and the process of ordering devices was done before the start of recruitment. Garmin device (the choice of wearable in this study) integration has been completed, and the device has been tested with three dummy Garmin accounts for data sanity checks. All documentation required for the study was completed and was approved for funding and sponsorship. Patient and public involvement (PPI) feedback on the app and schedule was arranged with a demonstration of the app and protocol to participants. Relevant changes were incorporated based on PPI feedback. The ethics application was submitted and final approval was obtained prior to the start of recruitment. Eight participants have been recruited in the ILD arm of the study, and one participant has been recruited in the COPD arm. Looking at the data dashboard, it is evident that two exacerbations have already been captured. The real-time exacerbation detection system has been developed, and most of the parts of the infrastructure have been deployed and tested. The test data have been processed and are ready to feed into the algorithm. Currently, algorithm development is in progress, and we are using analyses from a priori data sets [13] and results from similar studies to inform the design of our algorithm.

## Discussion

### Conclusion

The RALPMH study was meticulously planned with collaboration from clinical teams, technical teams, and data analysis teams to provide a reference infrastructure for the use of wearable data for monitoring lung diseases. It specifically involves information regarding the feasibility and acceptability of remote monitoring and the potential of real-time remote data collection and analysis in the context of chronic lung disorders. Moreover, it provides a unique standpoint to look into the specifics of the novel coronavirus without burdensome interventions. It will help plan and inform decisions in future studies that make use of remote monitoring in the area of respiratory health.

### Ethical and Regulatory Considerations

#### Assessment and Management of Risk

Details on risk assessment are provided in Table 4.

**Table 4:** Risk assessment.

Description of risk (indicate the level of likelihood: low/medium/high)	Risk priority (low, medium, high)	Risk owner	Proposed risk-mitigation measures
Data protection (eg, from intrusions)	Low	KCL^a^/SLAM^b^	Restrict access control to the data and use data sensitivity tiering. Encryption of data in transmission and at rest. Deidentification/pseudonymization once data are collected. Linked strong identifiers will be removed. Maintenance of software updates.
Threats to patient privacy	Low	KCL/SLAM	Data are handled with attention to deidentification and encryption. Higher risk data will typically be processed on edge devices with only aggregated data sent forward.
Patient fatigue with active or passive components of data collection	Medium	KCL/UCL^c^	Early engagement of acceptable burden levels to define expected tolerance levels. Opportunity to adapt the active data collection components in the course of the study.

^a^KCL: King’s College London.

^b^SLAM: South London and Maudsley NHS Foundation Trust.

^c^UCL: University College London.

#### PPI

This project was developed following discussions with patients and their families, who wanted to ensure that their lung disease could be safely monitored at home. Patients from IPF and COPD groups read a draft protocol in full, and their feedback was used to improve the final submitted protocol. Information for patients and the public will be posted on the Breathing Matters website [45], and the final results will be shared with the patients.

#### Protocol Compliance

A repeating form on REDCap will be used to log any contact with participants and document any deviations from the protocol. Notifications to the principal investigator or sponsor will be reported appropriately.

Significant deviation from the protocol or noncompliance may result in the removal of the participant from the study upon review.

Logged telephone contact will be made with participants throughout the course of follow-up if there is a loss of data stream from a device. The participant will be contacted by telephone to ensure compliance and correct use. These contacts will be recorded as evidence of feasibility and acceptability outcomes. In addition to the telephone call provided after the introductory training session, participants will receive a further call after 1 month to address any further concerns or questions.

#### Access to the Final Study Data Set

The full data set for analysis will be limited to the immediate research groups and to members who must additionally hold current contracts with King’s College London or University College London. The data analysis will be conducted in the appropriate data safe havens and university compute infrastructure.

A secondary pseudonymized (removing any potential identifiers, such as raw location and audio data, will be restricted to the locked primary data set), postprocessed, and deidentified set of derived features (eg, activity, HR, and sleep features) may be published for reference, benchmarking, and review as part of academic literature generated from this project.

### Limitations

The size of the cohorts and the duration of the study might not be enough to get accurate and expected results, but this is typical for most pilot studies, as we intend to figure out the correct size and duration of the study required for expected results, which can be further used to plan future longitudinal studies. This can be partly mitigated by attempting to recruit people who are at high risk of exacerbation.

### Dissemination Policy

Analysis and access to the primary data set will remain on secure King’s College London/University College London infrastructure and will be jointly owned by the co-investigator groups and institutions. The secondary deidentified and pseudonymized data set may be published by the project partners as part of analysis and publication dissemination activity, and this may include the use of repositories, such as Synapse [46], with the intended time aim of 12 to 18 months after the conclusion of data collection. Partners may be notified of the published results on the project website [47].

## Appendix

Multimedia Appendix 1 Data analysis algorithms and models.

Multimedia Appendix 2 CONSORT-eHEALTH Checklist (V 1.6.1).

## Abbreviations

AE-IPF: acute exacerbations of idiopathic pulmonary fibrosis
aRMT: Active Remote Monitoring Technology
CCLTE: Centers for Disease Control and Prevention COVID-19 long-term effects
COPD: chronic obstructive pulmonary disease
eCRF: electronic case report form
ERS: Exacerbation Rating Scale
GAD-7: Generalized Anxiety Disorder scale
HR: heart rate
ILD: interstitial lung disease
IPF: idiopathic pulmonary fibrosis
mHealth: mobile health
NHS: National Health Service
PCFS: post-COVID functional status
PHQ-8: Patient Health Questionnaire depression scale
PIS: participant information sheet
PPI: patient and public involvement
QoL: quality of life
RADAR: Remote Assessment of Disease and Relapse
RADAR-CNS: Remote Assessment of Disease and Relapse – Central Nervous System
RALPMH: Remote Assessment of Lung Disease and Impact on Physical and Mental Health
RFH: Royal Free Hospital
RHR: resting heart rate
SpO_2_: oxygen saturation
UCLH: University College London Hospital
WHO: World Health Organization

## References

[1] Drake TM, Docherty AB, Harrison EM, Quint JK, Adamali H, Agnew S, Babu S, Barber CM, Barratt S, Bendstrup E, Bianchi S, Villegas DC, Chaudhuri N, Chua F, Coker R, Chang W, Crawshaw A, Crowley LE, Dosanjh D, Fiddler CA, Forrest IA, George PM, Gibbons MA, Groom K, Haney S, Hart SP, Heiden E, Henry M, Ho L, Hoyles RK, Hutchinson J, Hurley K, Jones M, Jones S, Kokosi M, Kreuter M, MacKay LS, Mahendran S, Margaritopoulos G, Molina-Molina M, Molyneaux PL, O'Brien A, O'Reilly K, Packham A, Parfrey H, Poletti V, Porter JC, Renzoni E, Rivera-Ortega P, Russell A, Saini G, Spencer LG, Stella GM, Stone H, Sturney S, Thickett D, Thillai M, Wallis T, Ward K, Wells AU, West A, Wickremasinghe M, Woodhead F, Hearson G, Howard L, Baillie JK, Openshaw PJM, Semple MG, Stewart I, Jenkins RG, ISARIC4C Investigators (2020). Outcome of Hospitalization for COVID-19 in Patients with Interstitial Lung Disease. An International Multicenter Study. Am J Respir Crit Care Med.

[2] Ranjan Y, Rashid Z, Stewart C, Conde P, Begale M, Verbeeck D, Boettcher S, Dobson R, Folarin A, Hyve, RADAR-CNS Consortium (2019). RADAR-Base: Open Source Mobile Health Platform for Collecting, Monitoring, and Analyzing Data Using Sensors, Wearables, and Mobile Devices. JMIR Mhealth Uhealth.

[3] RADAR-base.

[4] Apache Avro. Wikipedia.

[5] King TE, Tooze JA, Schwarz MI, Brown KR, Cherniack RM (2001). Predicting survival in idiopathic pulmonary fibrosis: scoring system and survival model. Am J Respir Crit Care Med.

[6] Lung disease in the UK. British Lung Foundation.

[7] Collard HR, Ryerson CJ, Corte TJ, Jenkins G, Kondoh Y, Lederer DJ, Lee JS, Maher TM, Wells AU, Antoniou KM, Behr J, Brown KK, Cottin V, Flaherty KR, Fukuoka J, Hansell DM, Johkoh T, Kaminski N, Kim DS, Kolb M, Lynch DA, Myers JL, Raghu G, Richeldi L, Taniguchi H, Martinez FJ (2016). Acute Exacerbation of Idiopathic Pulmonary Fibrosis. An International Working Group Report. Am J Respir Crit Care Med.

[8] Richeldi L, du Bois RM, Raghu G, Azuma A, Brown KK, Costabel U, Cottin V, Flaherty KR, Hansell DM, Inoue Y, Kim DS, Kolb M, Nicholson AG, Noble PW, Selman M, Taniguchi H, Brun M, Le Maulf F, Girard M, Stowasser S, Schlenker-Herceg R, Disse B, Collard HR, INPULSIS Trial Investigators (2014). Efficacy and safety of nintedanib in idiopathic pulmonary fibrosis. N Engl J Med.

[9] Mikolasch TA, Garthwaite HS, Porter JC (2017). Update in diagnosis and management of interstitial lung disease. Clin Med (Lond).

[10] Management and Prevention of COPD. Global Initiative for Chronic Obstructive Lung Disease (GOLD).

[11] Hurst JR, Vestbo J, Anzueto A, Locantore N, Müllerova H, Tal-Singer R, Miller B, Lomas DA, Agusti A, Macnee W, Calverley P, Rennard S, Wouters EFM, Wedzicha JA, Evaluation of COPD Longitudinally to Identify Predictive Surrogate Endpoints (ECLIPSE) Investigators (2010). Susceptibility to exacerbation in chronic obstructive pulmonary disease. N Engl J Med.

[12] Hurst JR, Donaldson GC, Quint JK, Goldring JJP, Patel ARC, Wedzicha JA (2010). Domiciliary pulse-oximetry at exacerbation of chronic obstructive pulmonary disease: prospective pilot study. BMC Pulm Med.

[13] Al Rajeh AM, Aldabayan YS, Aldhahir A, Pickett E, Quaderi S, Alqahtani JS, Mandal S, Lipman MC, Hurst JR (2020). Once Daily Versus Overnight and Symptom Versus Physiological Monitoring to Detect Exacerbations of Chronic Obstructive Pulmonary Disease: Pilot Randomized Controlled Trial. JMIR Mhealth Uhealth.

[14] The Post-hospitalisation COVID-19 study (PHOSP-COVID). PHOSP.

[15] COVID-19. Centers for Disease Control and Prevention.

[16] Varatharaj A, Thomas N, Ellul MA, Davies NWS, Pollak TA, Tenorio EL, Sultan M, Easton A, Breen G, Zandi M, Coles JP, Manji H, Al-Shahi Salman R, Menon DK, Nicholson TR, Benjamin LA, Carson A, Smith C, Turner MR, Solomon T, Kneen R, Pett SL, Galea I, Thomas RH, Michael BD, Allen C, Archibald N, Arkell J, Arthur-Farraj P, Baker M, Ball H, Bradley-Barker V, Brown Z, Bruno S, Carey L, Carswell C, Chakrabarti A, Choulerton J, Daher M, Davies R, Di Marco Barros R, Dima S, Dunley R, Dutta D, Ellis R, Everitt A, Fady J, Fearon P, Fisniku L, Gbinigie I, Gemski A, Gillies E, Gkrania-Klotsas E, Grigg J, Hamdalla H, Hubbett J, Hunter N, Huys A, Ihmoda I, Ispoglou S, Jha A, Joussi R, Kalladka D, Khalifeh H, Kooij S, Kumar G, Kyaw S, Li L, Littleton E, Macleod M, Macleod Mj, Madigan B, Mahadasa V, Manoharan M, Marigold R, Marks I, Matthews P, Mccormick M, Mcinnes C, Metastasio A, Milburn-McNulty P, Mitchell C, Mitchell D, Morgans C, Morris H, Morrow J, Mubarak Mohamed A, Mulvenna P, Murphy L, Namushi R, Newman E, Phillips W, Pinto A, Price Da, Proschel H, Quinn T, Ramsey D, Roffe C, Ross Russell A, Samarasekera N, Sawcer S, Sayed W, Sekaran L, Serra-Mestres J, Snowdon V, Strike G, Sun J, Tang C, Vrana M, Wade R, Wharton C, Wiblin L, Boubriak I, Herman K, Plant G (2020). Neurological and neuropsychiatric complications of COVID-19 in 153 patients: a UK-wide surveillance study. The Lancet Psychiatry.

[17] COVID Collab.

[18] RADAR-CNS.

[19] Matcham F, Barattieri di San Pietro C, Bulgari V, de Girolamo G, Dobson R, Eriksson H, Folarin AA, Haro JM, Kerz M, Lamers F, Li Q, Manyakov NV, Mohr DC, Myin-Germeys I, Narayan V, Bwjh P, Ranjan Y, Rashid Z, Rintala A, Siddi S, Simblett SK, Wykes T, Hotopf M, RADAR-CNS consortium (2019). Remote assessment of disease and relapse in major depressive disorder (RADAR-MDD): a multi-centre prospective cohort study protocol. BMC Psychiatry.

[20] Shah SA, Velardo C, Farmer A, Tarassenko L (2017). Exacerbations in Chronic Obstructive Pulmonary Disease: Identification and Prediction Using a Digital Health System. J Med Internet Res.

[21] Ismail A, Musa A, Zim M, Fadzil M, Razali N (2016). Resting heart rate and exacerbations in COPD patients. European Respiratory Journal.

[22] Han MK, McLaughlin VV, Criner GJ, Martinez FJ (2007). Pulmonary diseases and the heart. Circulation.

[23] Yu C, Wong RS, Wu EB, Kong S, Wong J, Yip GW, Soo YOY, Chiu MLS, Chan Y, Hui D, Lee N, Wu A, Leung C, Sung JJ (2006). Cardiovascular complications of severe acute respiratory syndrome. Postgrad Med J.

[24] Chatterjee S, Rahman M, Nemati E, Nathan V, Vatanparvar K, Kuang J (2019). mLung++: automated characterization of abnormal lung sounds in pulmonary patients using multimodal mobile sensors. Adjunct Proceedings of the 2019 ACM International Joint Conference on Pervasive and Ubiquitous Computing and Proceedings of the 2019 ACM International Symposium on Wearable Computers.

[25] Wang X, Huang R, Mao S (2017). SonarBeat: Sonar Phase for Breathing Beat Monitoring with Smartphones.

[26] Jones SR, Carley S, Harrison M (2003). An introduction to power and sample size estimation. Emerg Med J.

[27] Activity Tracking Feature Explained. Garmin.

[28] What is Advanced Sleep Monitoring in Garmin Connect?. Garmin.

[29] Fatigue Severity Scale. Shirley Ryan AbilityLab.

[30] Approach S62 - Body Battery. Garmin.

[31] An Alternative Reference Index to Monitor Fatigue | Heart Rate Variability (HRV). Garmin.

[32] Activity Tracking and Fitness Metric Accuracy. Garmin.

[33] What is Heart-Rate Variability (HRV)?. Garmin.

[34] What Is the Respiration Rate Feature on My Garmin Device?. Garmin.

[35] Top FAQs for the Pulse Ox Feature on Garmin Watches. Garmin.

[36] Klok F, Boon GA, Barco S, Endres M, Geelhoed JJ, Knauss S, Rezek SA, Spruit MA, Vehreschild J, Siegerink B (2020). The Post-COVID-19 Functional Status scale: a tool to measure functional status over time after COVID-19. Eur Respir J.

[37] Coronavirus. World Health Organization.

[38] Jones PW, Harding G, Berry P, Wiklund I, Chen W, Kline Leidy N (2009). Development and first validation of the COPD Assessment Test. Eur Respir J.

[39] Jones P, Harding G, Wiklund I, Berry P, Leidy N (2009). Improving the process and outcome of care in COPD: development of a standardised assessment tool. Prim Care Respir J.

[40] Spitzer RL, Kroenke K, Williams JBW, Löwe B (2006). A brief measure for assessing generalized anxiety disorder: the GAD-7. Arch Intern Med.

[41] Kroenke K, Spitzer RL, Williams JB (2001). The PHQ-9: validity of a brief depression severity measure. J Gen Intern Med.

[42] Bartfield JM, Ushkow BS, Rosen JM, Dylong K (1994). Single Breath Counting in the Assessment of Pulmonary Function. Annals of Emergency Medicine.

[43] Dejonckere PH, Bradley P, Clemente P, Cornut G, Crevier-Buchman L, Friedrich G, Van De Heyning P, Remacle M, Woisard V, Committee on Phoniatrics of the European Laryngological Society (ELS) (2001). A basic protocol for functional assessment of voice pathology, especially for investigating the efficacy of (phonosurgical) treatments and evaluating new assessment techniques. Guideline elaborated by the Committee on Phoniatrics of the European Laryngological Society (ELS). Eur Arch Otorhinolaryngol.

[44] Scholkmann F, Wolf U (2019). The Pulse-Respiration Quotient: A Powerful but Untapped Parameter for Modern Studies About Human Physiology and Pathophysiology. Front Physiol.

[45] Fighting pulmonary fibrosis and infection. Breathing Matters.

[46] Synapse.

[47] Precision Health Informatics Data Lab.

