# Supplementary material for: Remote Assessment of Lung Disease and Impact on Physical and Mental Health (RALPMH): Protocol for a Prospective Observational Study
Source: JMIR Res Protoc. 2021 Oct 7;10(10):e28873. doi: 10.2196/28873 (PMC8500349; doi:10.2196/28873)
Supplement: Multimedia Appendix 1 [file resprot_v10i10e28873_app1.docx]

# **Multimedia Appendix 1: Data analysis algorithms and models**

| Method | Data Types | Characteristics |
| --- | --- | --- |
| Decision tree | Passive sensor data from Mobile and Wearables, location, active data | low complexity and high interpretability |
| Decision tables | Any type | Does not support hierarchies |
| KNN (K- Nearest Neighbour) | Passive sensor data from Mobile and Wearables | low complexity and high interpretability |
| Hidden Markov model (HMM) | Passive sensor data from Mobile and Wearables | Reliable, captures the transition among different types of activities, supports hierarchies |
| Support Vector Machines (SVM) | Any type | Hard to interpret, requires a binary class problem |
| AdaBoost | Passive sensor data from Mobile and Wearables | supports feature selection, good support for activity classification |
| Global iterative replacement (GIR) | Passive sensor data from Mobile and Wearables | For calculating optimal window size, small relative error with high computational efficiency |
| DeepSense | Passive sensor data from Mobile and Wearables, active data | Uses deep learning on multimodal sensor data, uses a combination of CNNs and RNNs |
| Least square Regression | Passive Wearable sensor data (like HR) | Needs precomputation of some variables, used with KNN for data imputation |
| DeepEar | Audio data | Uses Deep Boltzmann Machines (DBMs), works in diverse environments with background noise and at the edge (low power) |
| MFCC + SVM/CNN | Lung Audio Data | Uses data segmentation, cubic-spline interpolation, MFCC and SVM for detection of breathing rate and events(eg- wheezing) |

*Data analysis algorithms and models Analytical methods list*
